# The Case of a Lady at New York, North America, as Mentioned in Letter to a Friend, Dated May 23, 1801

**Published:** 1801-11

**Authors:** D. Cox


### *The Case of a Lady at New York, North America, as mentioned in Letter to a Friend, dated May* 23, 1801. *Communicated by* D. Cox, *Esq.*

I HAVE been greatly afflicted all laſt Winter with a complaint in my eyes of a ſingular nature; an inſtance of which none of the Phyſicians have ever heard or read of. After ſleeping an hour or two, on opening my eyes, which I cannot reſiſt,

---

fist, I am thrown into great agony; and this frequently happens four or five times in a night. My fight is very little affected by it, and there is little or no appearance of inflammation after I am thoroughly awake and during the day. It is thought to be in the eye-lid; and as I have tried every thing and found no relief, I begin to apprehend there is no cure to be expected, but one which will terminate my exiftence.

Note, The lady is upwards of fixty.

